# A simple dataset of water quality on aquaponic fish ponds based on an internet of things measurement device

**DOI:** 10.1016/j.dib.2023.109248

**Published:** 2023-05-18

**Authors:** Boby Siswanto, Yasi Dani, Doni Morika, Bubun Mardiyana

**Affiliations:** a Computer Science Department, School of Computer Science, Bina Nusantara University - Bandung Campus, Jakarta, Indonesia; b Interior Design Department, School of Design, Bina Nusantara University – Bandung Campus, Jakarta, Indonesia; c Maintenance Engineering Department, Building Management, Bina Nusantara University – Bandung Campus, Jakarta, Indonesia

**Keywords:** Aquaponic, IoT, esp8266, Urban farming, Fish ponds

## Abstract

This dataset contains pH, TDS, and water temperature measurements using internet of things devices and sensors. The dataset is collected using an IoT sensor with ESP8266 as the microcontroller. Urban farmers can use this dataset with limited land in aquaponic cultivation as initial reference values or novice researchers to implement basic machine learning algorithms. Measurements were made on aquaculture consisting of 1m^3^ pond media with a water volume of 1 m x 1 m × 70 cm and hydroponic media with the Nutrient Film Technique (NFT) system. Measurements were carried out for three months from January 2023 to March 2023. The available datasets are raw data and filtered data.

**Specifications Table**

**Table:** 

Subject	Agricultural Sciences
Specific subject area	Aquaculture
Type of data	Table
How the data were acquired	Data is collected in real-time using internet of things devices and sensors. Arduino IDE 1.8.5 is used with the C programming language. The sensors used are pH-4502C to measure water pH, DFROBOT Analog TDS Sensor to measure TDS values, DS18B20 to measure water temperature, and NodeMCU ESP8266 [1] as a microcontroller. The series of IoT devices were immersed in the pool from January 2023 to March 2023 for 24 h. The dataset has been cleaned of missing values and uploaded to the Kaggle Data Repository [2] and Mendeley Data [3], where it is licensed and free to download.
Data format	Raw and Filtered (in .csv format)
Description of data collection	Data was recorded from a 1m^3^ fish pond with a 1 m x 1 m x 70 cm volume containing 30 red tilapia measuring about 10 cm. Temperature, pH, and TDS values were measured for 24 h. Measurements were made in ponds that have aquatic plants on the surface; this is ignored. No water changes were performed during the measurement. The outside temperature of the pond and whether or not it rains are ignored.
Data source location	• Institution: Bina Nusantara University – Bandung Campus • City/Town/Region: Bandung • Country: Indonesia • Latitude and longitude (and GPS coordinates, if possible) for collected samples/data: −6.915120326895839, 107.59353953893746
Data accessibility	Repository name: Mendeley Data Data identification number: 10.17632/yd36bx6f8f.2 Direct URL to data: https://data.mendeley.com/datasets/yd36bx6f8f No access control is required to view or download the datasets
Related research article	N/A

## Value of the Data

• Complete data, no missing values with data dimensions of 118,286 rows and 5 columns (id, created_date, pH value, TDS, water temperature). There are two types of data, raw and filtered. The filtered data is based on the optimal value of the previously published reference values for each variable (pH, TDS, temp), pH data is in the range of 6.5–8.5 [4], TDS data <= 500 mg/L (ppm) [5], water temperature data ranges from 24 to 27 °C [6].
• This data can be used by novice researchers, students, and lecturers who need a simple dataset for basic research, for urban fish farmers who understand a little statistical data processing can process the data.
• This dataset can be used as material for analysis using statistical or machine learning techniques, such as correlation analysis, classification, clustering, and association rules mining.
• This dataset is simple, consists of 3 variable readings of the fish pond water quality, and has a time variable—readings from IoT devices in real-time for three months in early 2023, January – March 2023.
• Readings are done in real-time with the IoT sensor, PH-4502C to measure water pH, DFROBOT Analog TDS Sensor to measure TDS values, DS18B20 to measure water temperature, and NodeMCU ESP8266 as a microcontroller.
• Measurements were made on aquaculture consisting of a 1 m x 1 m x 1 m pond media with a water volume of 1 m x 1 m x 70 cm and hydroponic media with the Nutrient Film Technique (NFT) system. The pool is located at Binus University – Bandung campus, Indonesia, with coordinates latitude −6.915120326895839 and longitude 107.59353953893746.

## 1 Objective

This dataset was created to record the quality of pH, TDS, and water temperature in small fish ponds. Measurement of parameters in small ponds is intended to help urban farmers who have limited land to be able to cultivate aquaculture. Measurements are made by utilizing IoT tools to create an optimal network topology. Alternative options for using a microcontroller and how to send data to a database server in the cloud are selected. The microcontroller options are Arduino Uno, Raspberry Pi, and NodeMCU ESP-8266, while the delivery options are by Wi-Fi or GSM module. The NodeMCU ESP-8266 microcontroller was chosen because it has an integrated Wi-Fi module that sends data via a Wi-Fi network.

### 1.2 Previous Related Research

In a paper entitled A machine-learning-based IoT system for optimizing nutrient supply in commercial aquaponic operations written by Dhal, S.B. et al., setting concentrations of Calcium and ammonium using an IoT-based sensing and actuation system in a closed-loop set-up. These two parameters are determined to be adjusted based on the results of an analysis of a weekly basis dataset from three commercial aquaponic farms in Southeast Texas over a year, where dimension reduction and feature selection techniques are applied using machine learning. The test of this research was to compare the growth of tilapia fish and lettuce plants in 2 different pond conditions, namely in summer and winter. The study successfully implemented IoT systems combined with Machine Learning for optimizing nutrient supply in aquaponic solutions, where fish and lettuce plants grow better in summer [7].

Publication paper entitled An IoT-Based Data-Driven Real-Time Monitoring System for Control of Heavy Metals to Ensure Optimal Lettuce Growth in Hydroponic Set-Ups, written by Dhal, S.B. et al., containing research on the growth of lettuce plants in a hydroponics system. This research aims to create a low-cost real-time smart sensing and actuation system for controlling heavy metal concentrations in aquaponic solutions by implementing IoT and machine learning tools. The results of the research are known concentrations from the hydroponics environment without the need to bring them to the laboratory so that they can save time and money [8].

His published paper entitled Can Machine Learning classifiers be used to regulate nutrients using small training datasets for aquaponic irrigation?: A comparative analysis by Dhal, S.B. et al. aimed to investigate the possibility of using statistical techniques in the aquaponic domain. Variable data is obtained from reading IoT tools. The study analyzed 14 variables, namely Calcium (ppm), Potassium (ppm), Boron (ppm), Sulfate (ppm), Phosphorus (ppm), Conductivity, Iron (ppm), Zinc (ppm), Manganese (ppm), Charge Balance, Temperature (K), Humidity (%), Pressure (mm) and Precipitation (inch). Of the 14 variables, several variables were eliminated because they had a correlation value greater than 90%, namely Magnesium (ppm), Hardness (grains CaCO3/gallon), Hardness (ppm CaCO3), Alkalinity (ppm CaCO3), Total Dissolved Salts (ppm) and Copper (ppm). This study implemented statistical methods to predict the optimal nutrients required for fish and plant growth in a single aquaponic set-up [9].

Another study conducted by Dhal, S.B. et al., wrote in a paper entitled Nutrient optimization for plant growth in Aquaponic irrigation using Machine Learning for small training datasets. The study analyzed agricultural data in urban areas using statistical methods and machine learning on aquaponic cultivation. The variables analyzed were Calcium, magnesium, sodium, potassium, boron, carbonate, bicarbonate, sulfate, chloride, nitrates, potassium, pH, conductivity, Alkalinity, Total Dissolved Salts, and Sodium Adsorption Ratio. The results of this study are recommendations that aquaponic cultivation of plants and fish is good to do with the main variables that are taken into account are Potassium, Boron, Bicarbonate, Sulfate, and Chloride concentrations in the solution [10].

In 2022, research was carried out with the title Effects of long-term exposure to high temperature on growth performance, chemical composition, hematological and histological changes, and physiological responses in hybrid catfish. The research investigated the effect of high temperatures on the growth of catfish. The results are that catfish will grow more optimally if the water temperature is around 32 °Celcius [11].

## 2 Data Description

There are two datasets, namely raw data and filtered data with the extension comma-separated values (.csv), both of which have a table structure, as shown in Table 1. The raw data is pond_iot_2023_raw.csv, and the filtered data is pond_iot_2023.csv. Each table consists of 5 columns: id, created_date, water_pH, TDS, and water_temp.

**Table 1:** Fish pond dataset table characteristics.

Attribute	Descriptions	Filtered Data Range
Id	Integer	
created_date	Date Time	
water_pH	Float	6.5 - 8.5
TDS	Integer	0 - 500 mg/L (ppm)
water_temp	Float	24 - 27 °C

**Fig. 1:**
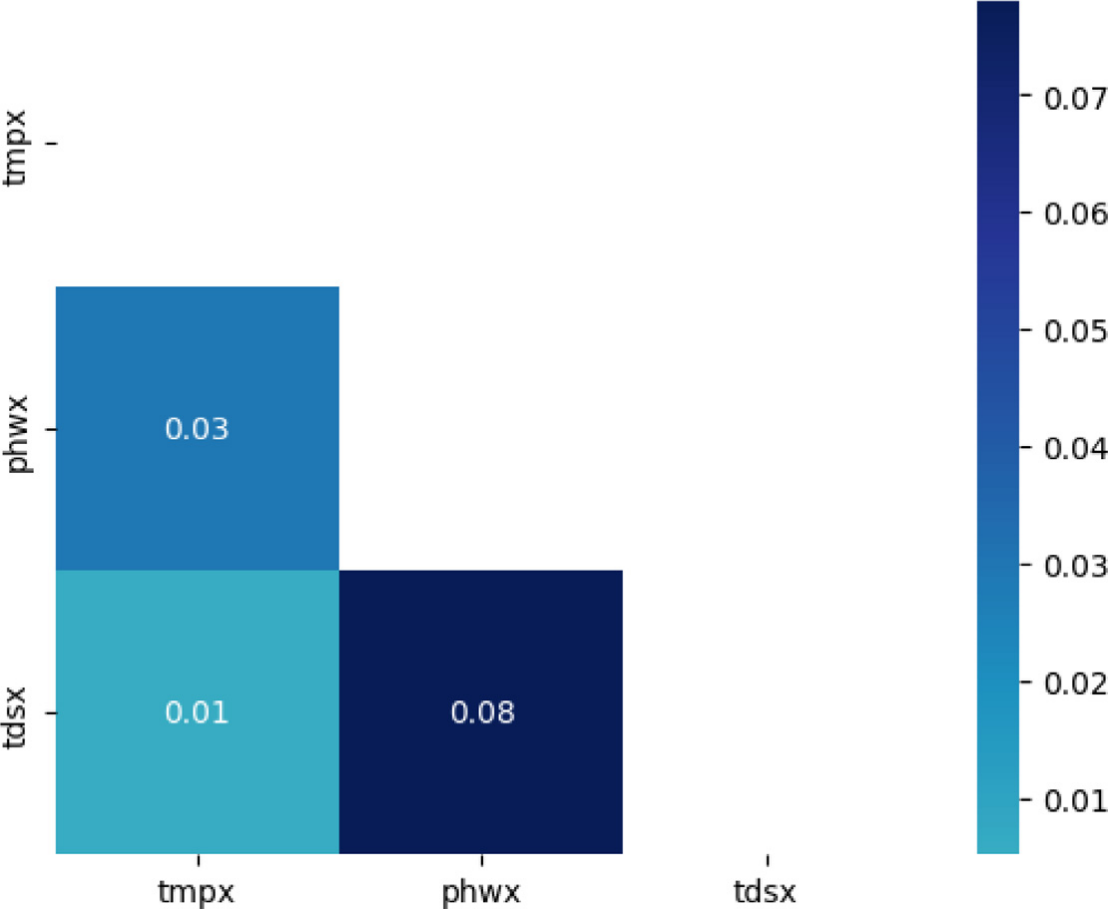
Screenshot of the simple IoT pond dataset bivariate correlation results.

The correlation of the dataset variables is shown in Fig. 1. You can see all the variables correlation values along with the color display of the data, the strength of the correlation value is shown in the histogram on the right. The darker the color indicates the stronger the correlation.

## 3 Experimental Design, Materials and Methods

**Fig. 2:**
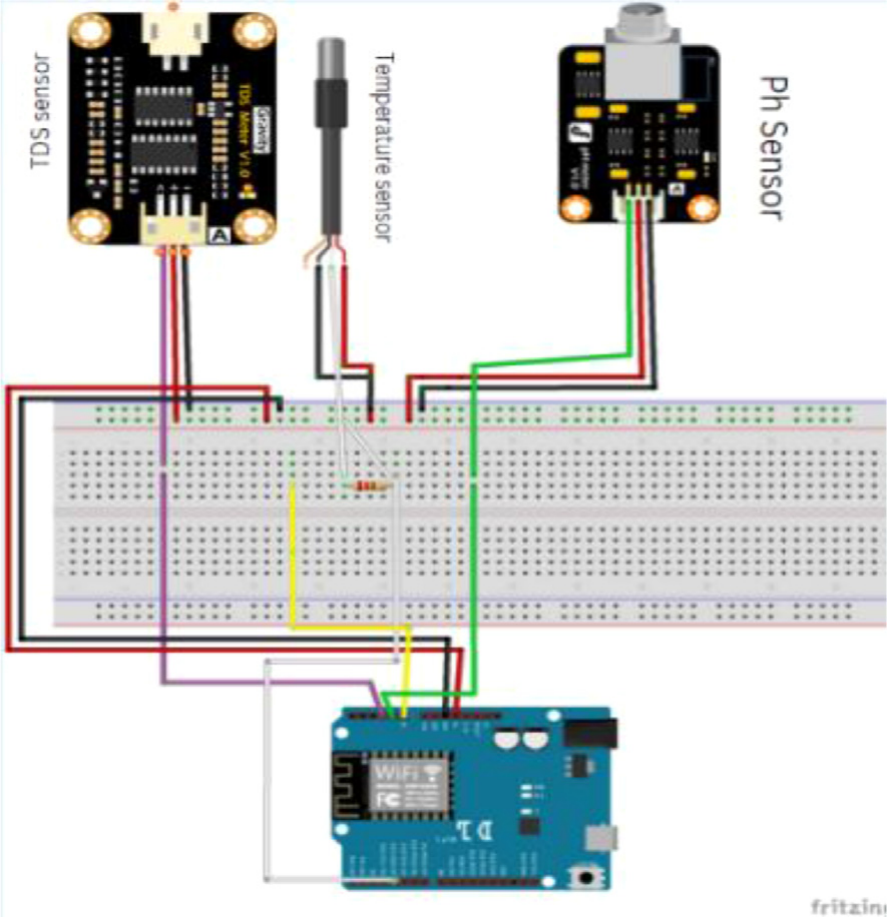
The schematic layout of an IoT device used in the data collection process.

This research implements IoT tools to monitor the quality of fish ponds. Three sensors and a microcontroller are used, as shown in Fig. 2, namely a pH sensor, a TDS sensor, and a water temperature sensor connected to the NodeMCU ESP-8266 microcontroller. The results of reading all sensors are sent to the online MySQL database in real-time via a Wi-Fi network.

**Fig. 3:**
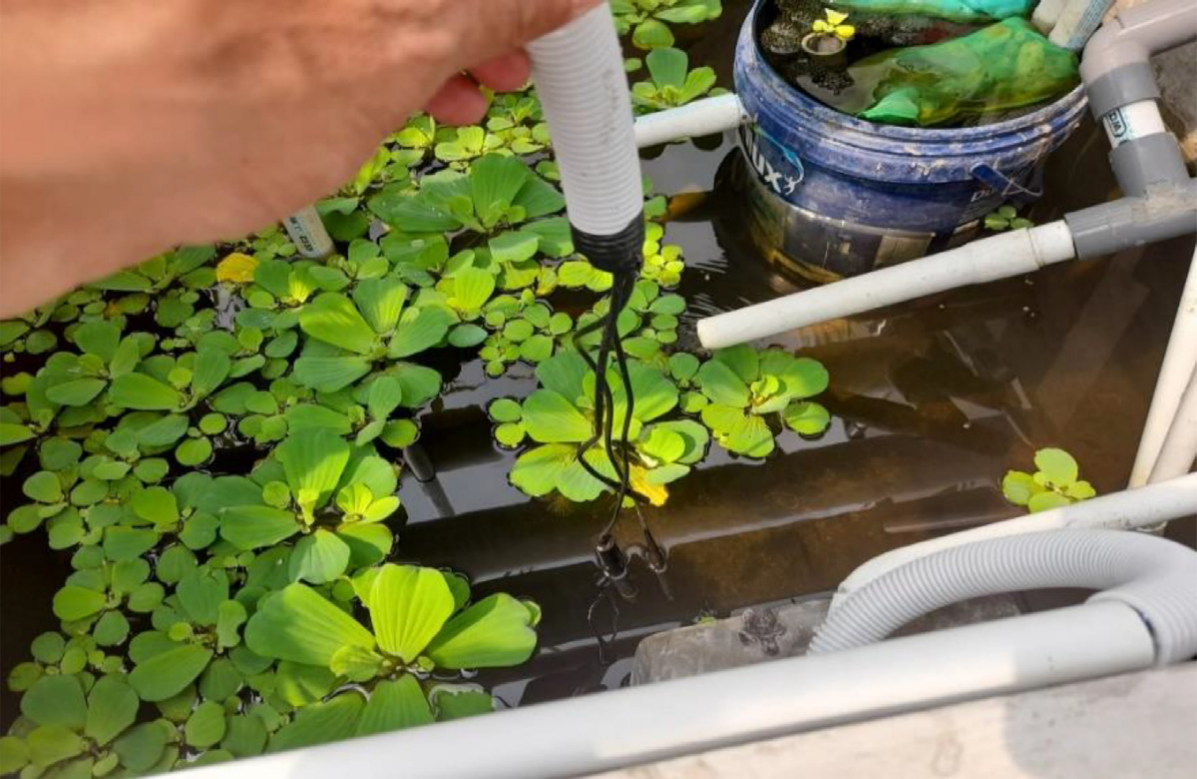
The pH sensor, TDS sensor, and temperature sensor.

**Fig. 4:**
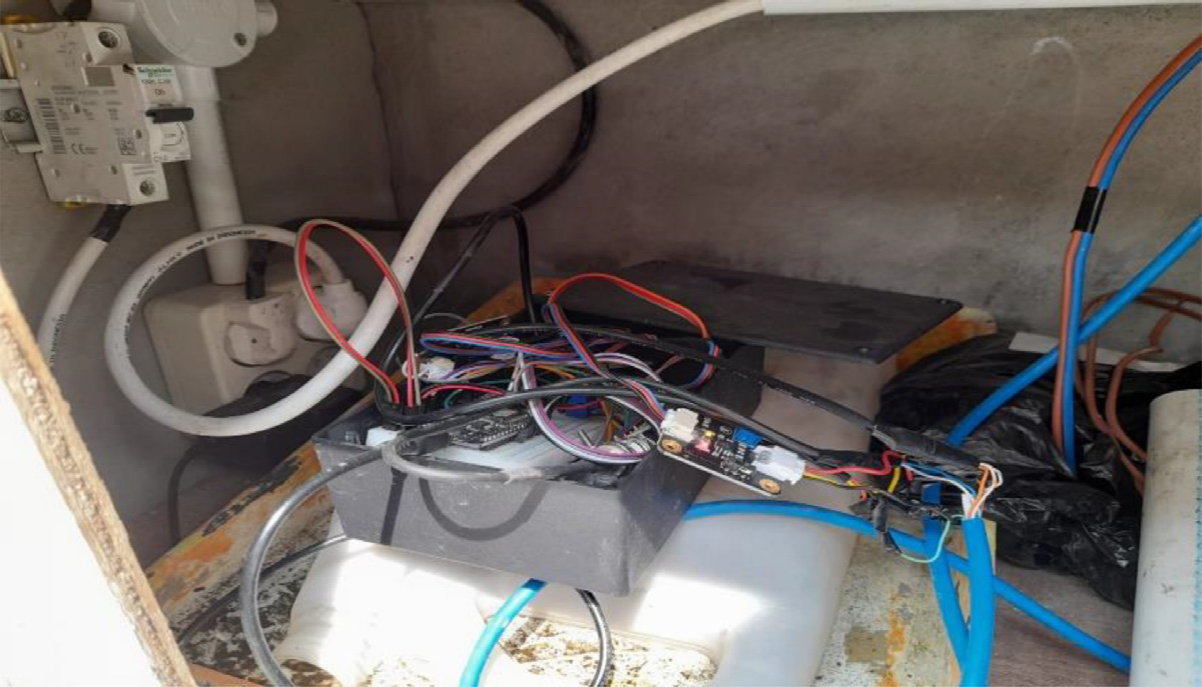
The connectivity of ESP-8266 with pH sensor, TDS sensor, and temperature sensor.

The three sensors are immersed in water at about 50 cm from the water's surface. The actual shape of the three sensors is shown in Fig. 3, with the tip of each sensor connected to the ESP-8266 microcontroller shown in Fig. 4.

**Fig. 5:**
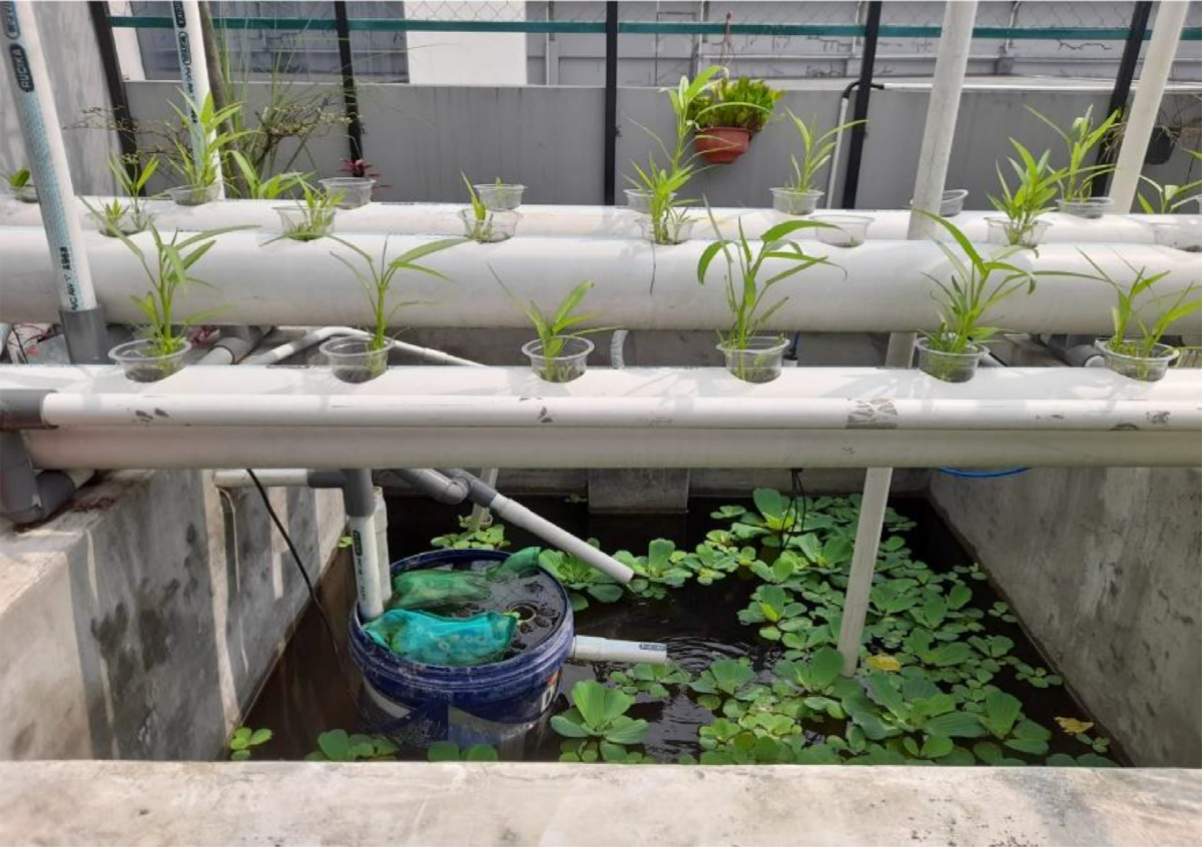
Environment visualization of the aquaponics system.

The research environment is shown in Fig. 5, where at the top are hydroponic plants whose water is connected via a pipe that is pumped up. The pool size is 1 m x 1 m x 1 m with a water volume of 1 m x 1 m x 70 cm. In the pond are 30 red tilapia fish with an initial size of about 10 cm.

## 4 Data Analysis

**Fig. 6:**
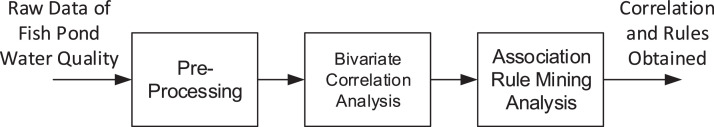
Data analysis diagram of the fish pond water quality.

Data analysis is carried out by implementing bivariate correlation analysis and association rule mining techniques to get the comparison between them. Correlation analysis results and the top five rules indicate the most strongly influencing water quality variables. Fig. 6 shows the flow of data analysis from the fish pond water quality dataset used.

The results of the bivariate correlation analysis are shown in Fig. 1, where there is a weak correlation between the three variables. Found correlations between temperature and pH of 0.03, a correlation between temperature and TDS of 0.01, and a correlation between pH and TDS of 0.08. Next are the results of the association rule analysis of the dataset, shown in Table 2. In the process of association rule mining, the values are grouped into three for each variable, namely low, mid, and high, with the reference value referring to Table 1. Temperature value group min is 24–25.9, mid is 26–26.9, and high is above 27. TDS min value group is 200–299, mid is 300–399, and high is above 400. pH min value group is 6.5–6.9, mid is 7–7.9, and high is above 8. It can be seen in Table 2 that if the TDS value is low, then the water temperature will also be low, and if the water pH value is high, which is above 8, then the temperature value will be in the range of 24–25.9 with a TDS value ranging at numbers 300–399.

**Table 2:** Association rules obtained on the fish pond dataset.

LHS	RHS	ConfValue
{tds_mid}	{tmp_low}	0.987548
{phw_high}	{tmp_low, tds_mid}	0.865411
{tmp_low}	{tds_mid}	0.732865
{phw_mid}	{tmp_low, tds_mid}	0.668631
{tmp_low}	{phw_mid}	0.540089

## 5 Conclusion

This dataset consists of two types of data files, raw data and filtered data, both of which contain readings of pH, TDS, and water temperature. The raw data of value comes from real-time recorded measurements of IoT data. The filtered data provided has been filtered according to the optimal recommended value, pH data is 6.5–8.5, TDS data <= 500 mg/L (ppm), and water temperature data ranges from 24 to 27 °Celcius.

## Ethics Statements

The experiment complied with the ARRIVE guidelines and was carried out according to the UK Animals (Scientific Procedures) Act, 1986, and associated guidelines; EU Directive 2010/63/EU for animal experiments.

## CRediT authorship contribution statement

**Boby Siswanto:** Conceptualization, Methodology, Software, Supervision, Writing – original draft. **Yasi Dani:** Data curation, Writing – review & editing. **Doni Morika:** Data curation, Investigation. **Bubun Mardiyana:** Data curation, Resources.

## Declaration of Competing Interest

The authors declare that they have no known competing financial interests or personal relationships that could have appeared to influence the work reported in this paper.

## Data Availability

A Simple Dataset of Aquaponic Fish Pond IoT (Original data) (Kaggle). A Simple Dataset of Aquaponic Fish Pond Water Quality Measurement using Internet of Things devices (Original data) (Mendeley Data).
